# Sleep, Sleepiness, and Alcohol Use

**Published:** 2001

**Authors:** Timothy Roehrs, Thomas Roth

**Affiliations:** Timothy Roehrs, Ph.D., is director of research at the Sleep Disorders and Research Center of the Henry Ford Hospital and adjunct professor of psychiatry in the Department of Psychiatry and Behavioral Neuroscience, Wayne State University, Detroit, Michigan. Thomas Roth, Ph.D., is division head of the Sleep Disorders and Research Center of the Henry Ford Hospital and adjunct professor of psychiatry in the Department of Psychiatry and Behavioral Neuroscience, Wayne State University, Detroit, Michigan

**Keywords:** sleep disorder, physiological AODE (effects of alcohol or other drug use, abuse, and dependence), REM (rapid eye movement) sleep, NREM (nonrapid eye movement) sleep, circadian rhythm, melatonin, prolactin, body temperature, attention, time of day, insomnia, dose-response relationship

## Abstract

The study of alcohol’s effects on sleep dates back to the late 1930s. Since then, an extensive literature has described alcohol’s effects on the sleep of healthy, nonalcoholic people. For example, studies found that in nonalcoholics who occasionally use alcohol, both high and low doses of alcohol initially improve sleep, although high alcohol doses can result in sleep disturbances during the second half of the nocturnal sleep period. Furthermore, people can rapidly develop tolerance to the sedative effects of alcohol. Researchers have investigated the interactive effects of alcohol with other determinants of daytime sleepiness. Such studies indicate that alcohol interacts with sleep deprivation and sleep restriction to exacerbate daytime sleepiness and alcohol-induced performance impairments. Alcohol’s effects on other physiological functions during sleep have yet to be documented thoroughly and unequivocally.

Alcohol affects sleep, daytime alertness, and certain physiological processes that occur during sleep. Its impact on human sleep has received much scientific study dating back to early experiments by Kleitman (1939), described in his book *Sleep and Wakefulness*. In that monograph, the author summarizes the effects that alcohol consumed 60 minutes before bedtime has on body temperature and motility during sleep in healthy nonalcoholic people. In the 1960s and 1970s, after scientists had identified various sleep states (e.g., rapid eye movement [REM] sleep) and had standardized electrophysiological methods to document sleep, research on alcohol’s effects on the sleep of healthy nonalcoholic and non-insomniac volunteers and on the sleep of alcoholics increased substantially. More recently, with the emergence of the field of sleep-disorders medicine, researchers and clinicians have focused their attention on alcohol’s effect on primary sleep disorders, such as sleep apneas, which are short (i.e., 10 to 30 seconds long) episodes of breathing obstruction. This attention to sleep disorders also has sensitized investigators and clinicians to the impact that disrupted and shortened sleep has on daytime alertness. As a result, various studies have investigated the potential interactive effects of alcohol with daytime alertness and daytime functioning in both healthy people and patients with sleep disorders.

This article provides an overview of alcohol’s effects on normal sleep, sleep physiology, and daytime alertness in nonalcoholic people. (The accompanying article by Brower, pp. 110–125 in this issue, discusses alcohol’s effects on sleep in alcoholics.) The current article reviews normal sleep physiology, describes alcohol’s effects on the various sleep states and sleep stages, and explores some of the mechanisms through which alcohol may exert those effects. It then summarizes the relationship of nocturnal sleep to daytime alertness and how alcohol affects this relationship. The article ends with a discussion of alcohol’s effects on sleep in people with primary insomnia.

## Normal Sleep Physiology

As most people know from their own experience, sleep is not uniform throughout the night. For example, at certain times during the night, it is very difficult to wake a sleeping person, whereas at other times, the slightest sound will alert the sleeper. Extensive studies have identified two different sleep states: REM sleep and nonrapid eye movement (NREM) sleep. Furthermore, NREM sleep can be divided into four stages based on how easy it is to arouse a sleeper (i.e., how “deep” the sleep is).

These different sleep states and sleep stages are defined based on scoring criteria for three electrophysiological measurements that were first published in 1968 and have been employed ever since in sleep laboratories around the world. The three electrophysiological measurements are recorded simultaneously and comprise the following:

- The electroencephalogram (EEG), which traces the electrical activity of the brain through electrodes placed on the scalp. These measurements produce characteristic brain waves called alpha, beta, delta, and theta rhythms, which differ in their frequencies.
- The electrooculogram (EOG), which measures eye movements through electrodes placed on the skin around the eyes and records tiny electric signals that occur when the eyes move.
- The electromyogram (EMG), which measures the electrical activity of muscles through electrodes placed on the skin in various body regions. This technique can measure even small muscle movement during sleep, such as twitching.

The following paragraphs describe how these measurements are used to distinguish different sleep states and sleep stages.

### Stages of NREM and REM Sleep

When comparing the EEG readings of various sleep stages, researchers and clinicians assess the frequency of the brain waves, measured in hertz (Hz), and the size, or amplitude, of the brain waves, measured in microvolts. Both the frequency and amplitude of the brain waves, as well as the EOG and EMG readings, differ for various stages of wakefulness and sleep (see figure).

During active wakefulness (i.e., when the person is awake and pursuing normal activities), the EEG is characterized by high frequencies (i.e., 16 to 25 Hz) and low voltage (i.e., 10 to 30 microvolts). EOG readings during wakefulness exhibit REMs, and EMG readings generally show a high amplitude indicative of large muscle movements.

During relaxed wakefulness (i.e., when a person is awake but has his or her eyes closed and is relaxed), the EEG is characterized by a pattern of alpha waves with a frequency of 8 to 12 Hz and an amplitude of 20 to 40 microvolts. EOG readings show slow, rolling movements at the transition to NREM sleep. EMG readings show reduced amplitudes.

During NREM sleep, the frequency of the brain waves slows further, whereas the amplitude continues to increase. Thus, when the arousal threshold is highest (i.e., sleep is “deepest”), the EEG shows slow-wave sleep with a frequency of 0.5 to 2.0 Hz and an amplitude of 75 microvolts or greater. EOG tracings indicate cessation of eye movements, and EMG readings are gradually reduced, even though episodic repositioning of the body and other motor events occur. Based on the simultaneous analysis of all three measurements, NREM sleep is classified into four stages that are characterized by increasing arousal thresholds. Thus, stage 1 (i.e., drowsy sleep) has the lowest arousal threshold; stage 2 (i.e., light sleep) is intermediate; and stages 3 and 4 (i.e., deep sleep), which collectively are also called slow-wave sleep (SWS), have the highest arousal threshold.

During REM sleep, cortical EEG readings revert to the low-voltage-mixed-frequency pattern seen during drowsy sleep. The EOG displays the bursts of rapid eye movements that give this stage its name. The EMG is reduced to its lowest level for the night. In fact, most major voluntary muscle groups are paralyzed, because certain nerve cells in the spinal cord (i.e., motor neurons) are not responding to nerve signals. Arousal thresholds in REM are relatively low, similar to NREM stages 1 or 2.

### Tonic and Phasic Periods of REM Sleep

REM sleep can be further subdivided into tonic and phasic periods. During the tonic periods, which account for the majority of REM sleep, muscle tone is decreased and the EEG is similar to that seen during stage 1 NREM sleep. These tonic periods are interrupted by intermittent phasic REM events. For example, the eye movements characteristic of REM sleep occur in bursts during these phasic periods, which are followed by the tonic periods of EOG quiescence. Coupled with the bursts of eye movements are phasic muscle twitches, typically involving peripheral muscles, although the reduced muscle tone (i.e., atonia) characteristic of the tonic periods continues in most muscle groups. In addition, bursts of activity occur during the phasic periods in body functions that are controlled by the autonomic nervous system^1^; these bursts of activity are reflected by irregularities in cardiopulmonary function (e.g., heart rate and breathing rate).

### NREM–REM Cycles

An ultradian process—a biorhythm with a cycle of less than 24 hours— within sleep controls the alternation between NREM and REM sleep throughout the night. This ultradian process creates cycles of NREM sleep followed by REM sleep that last approximately 90 to 120 minutes, yielding four to five such cycles over a standard 8-hour sleep period. In the first two of those cycles, slow-wave NREM sleep predominates, whereas the REM periods are generally quite short (i.e., 5 to 10 minutes). Conversely, in the last two or three cycles, REM sleep predominates, sometimes continuing uninterrupted for 30 to 40 minutes, and slow-wave NREM sleep is almost nonexistent. (The significance of this ultradian cycling of NREM and REM sleep to alcohol’s effects on sleep is described in the following section of this article.)

## Alcohol’s Effects on Sleep Physiology

To assess alcohol’s effects on sleep, investigators conducting a typical sleep study administer alcohol to their subjects approximately 30 to 60 minutes before bedtime. As a result of this schedule, alcohol concentrations in the breath or blood usually peak at “lights-out.” Using this approach, researchers have extensively studied alcohol’s effects in healthy people at doses ranging from 0.16 to 1.0 grams of alcohol per kilogram of body weight (g/kg) (Williams and Salamy 1972). These doses, which correspond to approximately one to six standard drinks,^2^ yield breath alcohol concentrations (BrACs) as high as 0.105 percent.^3^ Some studies using this range of alcohol doses reported that the study participants fell asleep faster (i.e., had reduced sleep latency) than without alcohol consumption. One study found an increased sleep time at a low alcohol dose (i.e., 0.16 g/kg) but detected no such effect at higher alcohol doses (i.e., 0.32 and 0.64 g/kg) (Stone 1980).

Some investigators have separately analyzed alcohol’s effects during the first and second half of the nighttime sleep period. These studies found that particularly at higher alcohol doses, increased wake periods or light stage 1 sleep periods occurred during the second half of the sleep period (Williams et al. 1983; Roehrs et al. 1991). This second-half disruption of sleep continuity is generally interpreted as a “rebound effect” once alcohol has been completely metabolized and eliminated from the body. The term “rebound effect” means that certain physiological variables (e.g., sleep variables, such as the amount of REM sleep) change in the opposite direction to the changes induced by alcohol and even exceed normal levels once alcohol is eliminated from the body. This effect results from the body’s adjustment to the presence of alcohol during the first half of the sleep period in an effort to maintain a normal sleep pattern. Once alcohol is eliminated from the body, however, these adjustments result in sleep disruption. This hypothesis is supported by the known rate of alcohol metabolism, which leads to a decrease in BrAC of 0.01 to 0.02 percent per hour. Given that in such experiments, the typical peak BrACs measured shortly before sleep are 0.06 to 0.08 percent, alcohol metabolism at this rate would be completed within 4 to 5 hours of sleep onset; thus, the sleep disruption during the second half of the night would coincide with the clearance of alcohol from the body.

In addition to these effects on sleep initiation and sleep maintenance, researchers have found that alcohol consistently affects the proportions of the various sleep stages. Thus, most studies have reported a dose-dependent suppression of REM sleep at least during the first half of the sleep period (Williams and Salamy 1972). As noted earlier, the amount of REM sleep time is lower during the first half of the night relative to the second half of the night; consequently, the full REM-suppressive effect of alcohol is probably underestimated in most studies. To determine alcohol’s full effect on REM sleep, investigators would need to administer an additional alcohol dose in the middle of the night, thereby causing alcohol’s peak concentrations to coincide with the majority of REM sleep time. No such studies have been conducted, however.

Those studies that have demonstrated alcohol-induced REM suppression during the first half of the sleep period also have frequently found an REM rebound (i.e., longer-than-normal REM periods) during the second half of the night (Williams and Salamy 1972). As a result, the overall amount of REM sleep in subjects receiving alcohol before sleeping did not differ from that in subjects receiving a nonalcoholic drink (i.e., a placebo). As with the increased periods of wakefulness or light sleep, the REM rebound during the second half of the night is associated with the completed alcohol metabolism and elimination from the body. The neurobiological mechanisms responsible for the rebound of either wakefulness or REM sleep are still unknown.

Some studies also found an alcohol-related increase in the amount of SWS (i.e., stages 3 and 4 NREM sleep) in the first half of the sleep period (Williams and Salamy 1972). In addition to the alcohol dose consumed, the basal (i.e., normal) level of SWS in the study population appeared to be the most likely factor determining whether SWS was increased. For example, in a study of insomniacs who had lower amounts of SWS than did healthy people when taking a placebo—a typical finding in insomniacs—SWS increased when they consumed alcohol (Roehrs et al. 1999). Conversely, alcohol did not affect SWS in a group of age-matched healthy control subjects.

Another population that typically shows lower levels of SWS compared with healthy young adults are the elderly, but no studies have assessed alcohol’s effects on the sleep of healthy elderly people. In sleep deprivation studies, however, elderly participants show increases in SWS on the recovery night after the sleep-deprivation period; possibly alcohol could similarly promote SWS in elderly people. This finding does not imply, however, that alcohol should be considered a potential sleep therapy in elderly people, because tolerance to the SWS enhancement develops rapidly (Prinz et al. 1980).

Several studies have assessed the effects of alcohol administration over several nights. Such studies clearly demonstrated that tolerance to alcohol’s sedative and sleep-stage effects develops within 3 nights (Williams and Salamy 1972) and that the percentages of SWS and REM sleep return to basal levels after that time. Furthermore, in some studies, the discontinuation of nightly alcohol administration resulted in a REM sleep rebound—that is, an increase in REM sleep beyond basal levels (Williams and Salamy 1972). However, not all studies found such a rebound effect. This variability in results may be related to several factors specific for each study, including the basal level of REM sleep in the participants, the degree of alcohol-related REM suppression, the extent of prior tolerance to REM suppression, and the dose and duration of alcohol administration.

## Alcohol’s Effects on Hormone Function

The sleep-wake cycle is organized in a circadian rhythm. To track this rhythm in humans, researchers tend to use measurements of the core body temperature and of the secretion of the hormone melatonin from the pineal gland in the brain, both of which fluctuate in a typical pattern throughout the day. Accordingly, one can also use these measurements to assess alcohol’s effects on the sleep-wake cycle. As noted earlier, Kleitman (1939) first reported that alcohol administration 60 minutes before nocturnal bedtime altered body temperature compared with placebo administration. Thus, alcohol administration initially resulted in a reduction in core temperature, followed by a rebound increase in temperature. Such a temperature-reducing (i.e., hypothermic) effect of alcohol also has been observed in numerous other studies.

Various hormones secreted by the pituitary gland in the brain also show circadian variations, with secretory peaks occurring during the usual sleep period. Some of these hormones are linked to sleep—if sleep is delayed, their secretory peaks also are delayed. Conversely, the levels of other hormones peak at the same time every night, even if sleep is delayed. One of the pituitary hormones linked to sleep is growth hormone, whose secretion typically peaks with the onset of SWS (Takahashi et al. 1969). In an early study, administration of 0.8 g/kg alcohol before bedtime suppressed growth-hormone secretion, despite increasing the percentage of SWS (Prinz et al. 1980). A later study using two different alcohol doses—0.5 and 1.0 g/kg—similarly found that alcohol suppressed growth-hormone secretion at a dose-related rate (Ekman et al. 1996). Thus, alcohol appears to affect growth-hormone secretion and SWS levels independently (i.e., to dissociate growth hormone from SWS).

This hypothesis is further supported by the results of repeated alcohol administration in the first study (Prinz et al. 1980). In that study, the alcohol-related suppression of growth-hormone secretion persisted over the 3 nights of alcohol administration, whereas tolerance developed to the alcohol-related enhancement of SWS. The clinical implications of alcohol’s inhibitory effects on growth hormone and the dissociation of growth hormone and SWS are unclear, particularly with chronic and excessive alcohol use. Unfortunately, these provocative findings have not been pursued further.

Another pituitary hormone linked to sleep is prolactin^4^; the hormone’s secretion peaks 4 to 5 hours after sleep onset (Van Cauter and Turek 1994). To date, researchers have not determined conclusively whether alcohol affects prolactin release. In the study by Ekman and colleagues (1996), alcohol did not affect prolactin levels. However, possibly even at the 1.0 g/kg alcohol dose, alcohol levels may no longer have been high enough 4 to 5 hours after sleep onset to affect prolactin secretion. Prinz and colleagues (1980) did not measure prolactin levels in their study.

## Alcohol’s Effects on Neurochemicals

Alcohol’s effects on central nervous system (CNS) function are mediated by its effects on various brain chemicals (i.e., neurotransmitters and neuromodulators) that are responsible for the transmission of nerve signals from one nerve cell (i.e., neuron) to the next. These neurotransmitters are released by the signal-emitting neuron and generally exert their actions by interacting with certain molecules (i.e., receptors) located on the surface of the signal-receiving neuron. Particularly at low doses, alcohol affects CNS function primarily by interfering with the normal actions of the neurotransmitters gamma-aminobutyric acid (GABA) and glutamate, both of which also play critical roles in wake-sleep states (Koob 1996).

GABA is the major inhibitory neurotransmitter system in the CNS—that is, its interaction with the signal-receiving neuron dampens the ability of that neuron to generate a new nerve signal. Evidence from studies using various types of experimental approaches has indicated that alcohol at low doses enhances GABA’s actions on the signal-receiving neuron, thereby reducing that neuron’s ability to generate nerve signals even further (Mihic and Harris 1996). This observation is significant, because many hypnotic drugs (i.e., barbiturates, benzodiazepines, and the newer non-benzodiazepine GABA agonists^5^) also act by facilitating GABA function. Scientists have long considered GABA to play a major role in sleep (Jones 2000). For example, GABA-releasing neurons are present in various brain areas that are involved in the generation of SWS, such as the brainstem reticular activation system, thalamus, hypothalamus, and basal forebrain. Thus, facilitation of GABA-mediated inhibition is one possible explanation for alcohol’s sedative and SWS-promoting effects.

Glutamate is the major excitatory neurotransmitter in the CNS—that is, the interaction of glutamate with its receptor activates the signal-receiving neuron to generate a new nerve signal. Four types of glutamate receptors have been identified, including the NMDA receptor (Tabakoff and Hoffman 1996). Anatomically, glutamate-releasing neurons also are present in some of the brain areas that promote SWS, such as the reticular activating system of the brainstem and the forebrain (Jones 2000). NMDA agonists produce seizures; conversely, some glutamate antagonists^6^ are used as sedatives and anesthetics (Jones 2000). Thus, glutamate is an important element in wakefulness and activation. Numerous biochemical and electrophysiological studies have found that alcohol inhibits NMDA-receptor function, thereby acting as a glutamate antagonist (e.g., Tabakoff and Hoffman 1996). Consequently, alcohol inhibition of NMDA function may be another mechanism through which alcohol derives its sedative effects.

In addition to GABA and the glutamate-NMDA system, another agent that only recently has been considered a candidate for mediating alcohol’s sleep effects is adenosine. This molecule is not a neurotransmitter itself but modulates signal transmission by other neurotransmitters, including GABA and glutamate. In general, adenosine inhibits the function of glutamate in the CNS (Dunwiddie 1996). Alcohol appears to facilitate these inhibitory modulatory effects of adenosine through several mechanisms, such as enhancing the formation of adenosine; inhibiting the return of released adenosine into the cells, thereby prolonging its actions; and enhancing adenosine-receptor function (Dunwiddie 1996). Adenosine has been hypothesized to function as the sleep homeostat—the system that monitors the accumulated amount of wakefulness and sleep and signals the need for sleep (Bennington and Heller 1995). Its levels in the brain rise during waking and decline during SWS. Thus, alcohol also may promote SWS and rapid sleep onset by facilitating adenosine function.

The neurobiological mechanism underlying alcohol’s suppression of REM sleep is unclear. One neurotransmitter considered to play an important role in REM sleep is acetylcholine (Bennington and Heller 1995). Like other neurotransmitters, this molecule acts through several types of receptors, including nicotinic receptors and muscarinic receptors. To date, only minimal evidence suggests a substantive alcohol effect on acetylcholine. Furthermore, the evidence that does exist indicates that alcohol’s effects occur through the nicotinic acetylcholine receptor (Collins 1996); however, acetylcholine-mediated induction of REM sleep occurs through muscarinic receptors (Bennington and Heller 1995). Thus, it appears unlikely that the alcohol-related suppression of REM sleep is mediated by alcohol’s effects on the acetylcholine system.

Glutamate also is involved in the induction of some REM sleep phenomena (Bennington and Heller 1995), and alcohol’s inhibition of glutamate was noted earlier in this article (Tabakoff and Hoffman 1996). However, alcohol does not appear to exert its sedative and REM-suppressive effects through the same mechanism (e.g., glutamate inhibition), because both effects can be experimentally dissociated. For example, in a recent report, caffeine reversed alcohol’s sedative effects but not its REM suppressive effects^7^ (Turner et al. 2000). In sum, alcohol’s REM-suppressive effects may occur through glutamate-related mechanisms, whereas its sedative effects occur through GABA-related mechanisms.

## Relation of Nocturnal Sleep to Daytime Alertness

As mentioned earlier, the identification and recognition of sleep disorders have sensitized clinical researchers to the importance of sleep quantity and continuity for optimal daytime alertness and performance. In healthy people, even relatively minimal (i.e., 1 to 3 hours) reductions in nocturnal sleep time for a single night can reduce alertness and performance efficiency during the following day. Moreover, these effects can accumulate across nights (Roehrs et al. 2000*a*). Similarly, a disruption of sleep continuity by auditory stimuli, without reductions in overall sleep time, results in reduced alertness and performance efficiency in healthy people (Roehrs et al. 2000*a*). This fragmentation of sleep continuity is characterized by increased amounts of stage 1 sleep and brief awakenings.

Several studies have evaluated next-day performance and alertness in healthy people who consumed alcohol before bedtime. In one study, young pilots drank alcohol between 6 p.m. and 9 p.m. in quantities sufficient to result in blood alcohol concentrations (BACs) of 0.10 and 0.12 percent right before bedtime. The following morning, more than 14 hours after consuming alcohol and with BACs at 0, the performance of pilots in a flight simulator was impaired relative to their performance after consuming a placebo (Yesavage and Leirer 1986).

To investigate whether alcohol-induced sleep disruption contributed to subsequent performance impairment, Roehrs and colleagues (1991) administered alcohol to healthy people before sleep, recorded their sleep, and assessed the participants’ alertness and performance throughout the following day. The alcohol doses used resulted in a BrAC of 0.06 percent before sleep. The study found that this dose was associated with an increase in the amount of stage 1 sleep in the second half of the night. The next day, the investigators assessed alertness using the Multiple Sleep Latency Test (MSLT), a reliable and well-validated electrophysiological test. Performance was evaluated with tests of auditory vigilance, in which the participants had to respond to a certain sound, or divided attention tasks, in which the participants had to perform two tasks simultaneously (Roehrs et al. 2000*a*). The study found that in the alcohol-consuming participants, next-day alertness as measured by the MSLT was reduced and divided-attention performance was impaired (Roehrs et al. 1991), demonstrating that alcohol can indirectly impair daytime alertness and performance through its disruptive effects on sleep. These reductions in alertness and performance were relatively minor in terms of percentage of the baseline values; in the performance of difficult tasks (e.g., driving a car or flying an airplane), however, even such minor impairments might have significant consequences.

### Direct Alcohol Effects on Daytime Alertness

Although alcohol generally is classified as a depressant drug, in fact it has both sedative and stimulatory effects. These differential (i.e., biphasic) effects are dependent on the alcohol dose consumed and on the phase of the BAC (Pohorecky 1977). Thus, stimulatory effects are evident primarily at low-to-moderate alcohol doses and when BACs ascend to a peak. Conversely, alcohol’s sedative effects occur at higher alcohol doses and when BACs decline. Nighttime sleep studies that demonstrated alcohol’s sedative effects (i.e., reduced sleep latencies) in healthy people typically used alcohol doses that resulted in BrACs above 0.05 percent (Williams and Salamy 1972). Furthermore, the alcohol generally was administered 30 to 60 minutes before sleep, thus allowing for alcohol concentrations to peak before bedtime. In other studies that also were conducted during the descending BAC phase, alcohol reduced sleep latency, as measured by a standard MSLT, and impaired both attention and reaction-time performance in a dose-dependent manner. These impairing effects persisted for at least 2 hours after the alcohol had been completely metabolized as evidenced by BrACs of 0 (Roehrs and Roth 1998).

Only one daytime study using a modified MSLT assessed alcohol’s sleep effects during both the ascending and descending phase of the BrACs. That study found increased sleep latencies at peak BrACs relative to placebo, consistent with alcohol’s stimulatory effects under these conditions (Papineau et al. 1988). During the subsequent descending phase of the BrACs, however, sleep latencies were reduced relative to placebo, confirming alcohol’s biphasic effects.

A series of studies explored the modulation of alcohol’s daytime sedative and performance-disrupting effects by a person’s basal level of sleepiness (Roehrs and Roth 1998). In these studies, the investigators first either shortened or extended the participants’ scheduled nocturnal sleep time and then administered alcohol doses of 0.4 to 0.8 g/kg the following day. Subsequently, the researchers assessed the participants’ levels of sleepiness or alertness as well as psychomotor performance for approximately 8 hours. The results indicated that the level of sleepiness or alertness at the time of alcohol administration altered alcohol’s subsequent sedating and performance-disrupting effects. Thus, increased sleepiness compounded alcohol’s effects, whereas increased alertness diminished alcohol’s effects. Furthermore, the investigators observed those effects whether they compared sleepy versus alert healthy people, whether they studied the same person before and after both sleep restriction and sleep extension, or whether they studied the same person at various times of the day when the levels of sleepiness are known to differ according to the typical circadian rhythm.

## Relationships Between Nocturnal Sleep, Daytime Alertness, and Alcohol-Consumption History

Until now, this article has explored alcohol’s effects on nocturnal sleep and daytime alertness. The relationship between sleepiness-alertness and alcohol consumption, however, may be bidirectional. Thus, some survey and laboratory data suggest that variations in the duration of nocturnal sleep and level of daytime sleepiness may play an important role in modulating alcohol consumption. For example, a British survey found a negative correlation between sleep times and alcohol consumption in men— that is, shorter periods of sleep were associated with heavier drinking (Palmer et al. 1980). Similarly, in a U.S. study of young adults, participants who reported needing only 6 hours of sleep or less had an earlier age of drinking onset and drank more per month than did participants who needed more sleep (Schuckit and Bernstein 1981), leading the investigators to hypothesize that short sleep is associated with heavier alcohol intake.

Laboratory studies of alcohol and mood have identified some interesting relations between daytime sleepiness-alertness and drinking. In such studies, the participants’ preference for alcohol is studied by offering them several beverage choices presented in color-coded cups in which the participants do not know which of the cups contain an alcoholic beverage. After the participants have tasted each beverage, they can choose which beverage they prefer. Using this procedure, de Wit and colleagues (1987, 1989) found that moderate drinkers who preferred an alcohol dose of 0.5 g/kg, which corresponds to approximately three drinks, in the laboratory tests felt less alert at that time than did drinkers who did not prefer alcohol. Furthermore, participants who preferred alcohol in those studies generally experienced alcohol as increasing their elation and vigor, whereas participants who did not prefer alcohol generally experienced alcohol as increasing their sleepiness.

In an alcohol challenge study, in which healthy young men received a certain alcohol dose, the men’s drinking histories predicted their subjective responses to alcohol (Schuckit and Klein 1991). Those participants with histories of greater alcohol consumption showed less self-rated sleepiness after the alcohol challenge than did participants with histories of lower alcohol consumption. Researchers do not know whether these individual differences in response to alcohol reflect different physiological states (i.e., whether people are actually more or less sleepy) or differences in the perception of a common physiological state (i.e., whether all people experience the same physiological state but differ in whether they perceive that state as “being sleepy”). In the latter case, the different perceptions of alcohol’s effects may result from differential expectations regarding alcohol’s effects.

## Alcohol’s Effects on the Sleep of Insomniacs

Approximately 10 to 15 percent of the U.S. general population experiences difficulties falling asleep or maintaining sleep, or suffer from nonrestorative sleep (i.e., sleep that does not result in a feeling of being rested) (Roehrs et al. 2000*b*). Moreover, 30 percent of people with persistent insomnia in the general population have reported using alcohol to help them sleep in the past year, and 67 percent of those people have reported that alcohol was effective in inducing sleep (Ancoli-Israel and Roth 2000).

For several reasons, studies conducted in healthy people sleeping at their usual bedtimes, such as the studies reviewed in this article, do not adequately represent the hypnotic potential of alcohol in people with insomnia. First, in healthy people, sleep latency and sleep efficiency are already optimal, and further improvement is difficult to demonstrate. Consequently, as previously noted, alcohol’s effects on measures of sleep induction and maintenance in healthy people are minimal and inconsistent. Second, the doses used in sleep studies are generally much larger (i.e., resulting in BrACs greater than 0.05 percent, which corresponds to more than three drinks) than the doses that insomniacs typically report using (i.e., one to two drinks). Third, the same alcohol dose may have different effects in healthy people and insomniacs. A recent study compared the effects of an alcohol dose of 0.5 g/kg on the sleep of insomniacs and age-matched healthy people (Roehrs et al. 1999). In the insomniacs, but not in the healthy control subjects, this alcohol dose improved sleep compared with a placebo. Furthermore, the sleep disruption during the second half of the night that occurs in healthy people after higher alcohol doses was not observed in the insomniacs. Specifically, alcohol consumption in the insomniacs increased their SWS to the levels of the age-matched control subjects.

During a later phase of the same study (Roehrs et al. 1999), the participants also had an opportunity to choose between beverages presented in color-coded cups that contained various alcohol concentrations or a placebo. The participants had previously experienced all of those beverages (i.e., they had taken them one at a time before bedtime on different nights) and were asked to choose the beverage that would best help them sleep. With this approach, the insomniacs generally chose an alcohol-containing beverage, whereas the healthy people chose the placebo-containing beverage. Furthermore, the average nightly alcohol dose self-administered by the insomniacs was 0.45 g/kg (up to 0.6 g/kg was possible), which is similar to the dose previously shown to improve the sleep of the insomniacs and similar to the dose that insomniacs report using at home.

The epidemiological data and laboratory study findings indicating the preference for alcohol at bedtime by insomniacs, compared with noninsomniacs, generate several questions. For example, does this preference reflect the use of alcohol as self-medication for a sleep problem, as a way to improve mood, or as a sleep medication that subsequently becomes a “mood-altering” drug? And if alcohol use initially is, or ultimately becomes, “mood-altering” behavior, what are the “mood-altering” effects for the insomniac that reinforce alcohol consumption? Furthermore, do insomniacs develop tolerance to alcohol’s sedative effects as do other people? Do insomniacs increase their alcohol dose in successive nights? Does hypnotic use at night generalize to daytime use? And ultimately, what are the risks associated with the use of alcohol as a hypnotic? All these issues have yet to be addressed. But these data again suggest that the alcohol-sleep relation is interactive— that is, disturbed nocturnal sleep increases the likelihood of alcohol use, and alcohol has the potential to influence sleep.

## Summary

Alcohol has extensive effects on sleep and daytime sleepiness. In healthy people, acute high alcohol doses disturb sleep, whereas in insomniacs, lower doses may be beneficial. Data from healthy people suggest, however, that tolerance to alcohol’s sedative effects probably develops rapidly. This tolerance development may lead to excessive hypnotic use and, possibly, excessive daytime use for insomniacs. The effects of alcohol appear to be bidirectional in that nocturnal sleep quantity and continuity and subsequent levels of daytime sleepiness also influence alcohol’s sedative and performance-impairing effects. Sleep quality and daytime sleepiness may also relate to rates of alcohol drinking and become a gateway to excessive alcohol use. To investigate these issues and identify the mechanisms underlying the relationship between alcohol and sleep remain important tasks, as does documenting alcohol’s effects on other physiological functions during sleep.

## Figures and Tables

**Figure:**
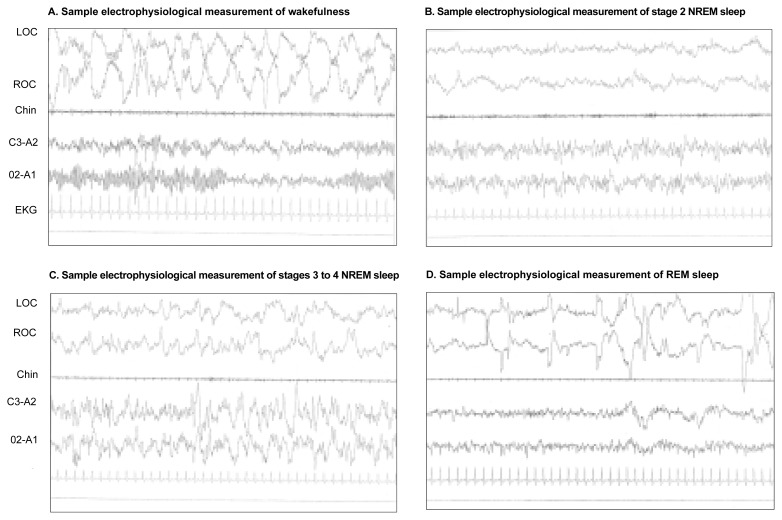
Samples of electrophysiological measurements of various sleep stages. The four panels represent the measurements obtained during (A) wakefulness; (B) stage 2 nonrapid eye movement (NREM) sleep (i.e., light sleep); (C) stages 3 to 4 NREM sleep (deep or slow-wave sleep); and (D) rapid eye movement (REM) sleep, which is associated with dreaming. For each panel, the graphs labeled LOC and ROC represent measurements of the left and right eye movements, respectively. The graph labeled “chin” represents a measurement of small body movements, such as of the chin muscles. The graphs labeled C3-A2 and O2–A1 represent two electroencephalogram (EEG) readings measuring brain activity in certain brain regions. Finally, the electrocardiogram (EKG) measures the heart rate. Each sleep stage is characterized by a specific pattern of those readings. For example, during REM sleep the eyes move rapidly compared with stage 2 NREM sleep. At the same time, the EEG readings during REM sleep exhibit a higher frequency (i.e., number of waves per second) and a lower amplitude (i.e., height of the peaks and valleys of the waves) compared with stage 2 NREM sleep.

## Footnotes

1 The autonomic nervous system controls involuntary vital functions, such as the activities of the heart, lungs, gastrointestinal tract, and glands.

2 A standard drink is defined as one 12-ounce bottle of beer or wine cooler, one 5-ounce glass of wine, and 1.5 ounces of 80 proof distilled spirits.

3 Breath alcohol concentrations are another way of quantifying alcohol levels in the body and are approximately the same as blood alcohol concentrations after a given alcohol dose.

4 Together with other hormones, prolactin regulates growth and development of the mammary glands and tinitiation and maintenance of milk production in nursing women.

5 Agonists are substances that mimic the actions of another molecule. For example, GABA agonists cause the same reactions in other neurons as does GABA.

6 Antagonists are substances that inhibit or interfere with the actions of another molecule. For example, glutamate antagonists inhibit glutamate’s interactions with its receptors.

7 Although unlikely at the low dose used, caffeine’s own REM-suppressive effects may have been responsible for the REM suppression observed.
